# Automated Differentiation of Oral Red‐White Lesions: An Interpretable Deep Learning Approach Combining Ensemble Architectures and Saliency Maps

**DOI:** 10.1002/cre2.70420

**Published:** 2026-07-21

**Authors:** Mahsa Koochaki, Amirreza Mousavie, Maryam Basirat, Amirreza Hendi, Hossein Sadr, Mojdeh Nazari

**Affiliations:** ^1^ Department of Oral and Maxillofacial Medicine, School of Dentistry Guilan University of Medical Sciences Rasht Iran; ^2^ Student Research Committee, School of Dentistry Guilan University of Medical Sciences Rasht Iran; ^3^ Department of Prosthodontics, Dental Sciences Research Center, School of Dentistry Guilan University of Medical Sciences Rasht Iran; ^4^ Neuroscience Research Center, Trauma Institute Guilan University of Medical Sciences Rasht Iran; ^5^ Department of Artificial Intelligence in Medicine, Faculty of Advanced Technologies in Medicine Iran University of Medical Sciences Tehran Iran; ^6^ Department of Cardiology, Cardiovascular Disease Research Center, Heshmat Hospital, School of Medicine Guilan University of Medical Sciences Rasht Iran; ^7^ Department of Health Information Technology and Management, School of Allied Medical Sciences Shahid Beheshti University of Medical Sciences Tehran Iran

**Keywords:** deep learning, early diagnosis, explainable AI, oral lichen planus, oral potentially malignant disorders, weighted ensemble

## Abstract

**Introduction:**

Oral Potentially Malignant Disorders (OPMDs), including Leukoplakia and Erythroplakia, carry significant risks of malignant transformation. Early differentiation from confounding inflammatory conditions like Oral Lichen Planus (OLP) and Candidiasis is critical yet challenging due to visual similarities. This study develops a robust, interpretable deep learning framework for automated multi‐class classification of pre‐localized oral lesions.

**Materials and Methods:**

A curated dataset of 705 high‐resolution images across five categories (Leukoplakia, Erythroplakia, OLP, Candidiasis, and Normal) was utilized. We proposed a Multi‐Architecture Weighted Ensemble Framework integrating ResNet‐50, Xception, and EfficientNet‐B0. A stratified patient‐level splitting strategy prevented data leakage. Model interpretability and clinical utility were assessed via Gradient‐weighted Class Activation Mapping (Grad‐CAM) and Decision Curve Analysis (DCA).

**Results:**

The ensemble model achieved 91.2% accuracy and a 90.8% macro‐averaged F1‐score, significantly outperforming individual baselines. The strategy improved OLP detection (F1‐score: 0.83), effectively distinguishing it from Leukoplakia. Grad‐CAM confirmed the model focuses on pathognomonic lesion features rather than confounding artifacts. DCA suggested a potential theoretical net clinical benefit over default strategies.

**Conclusion:**

This weighted ensemble framework demonstrates high retrospective accuracy and provides transparent visual explanations for the classification of pre‐localized oral lesions. However, it must be interpreted strictly as a preliminary proof‐of‐concept investigation. While the current results suggest potential adjunctive value, extensive external validation, prospective testing, and clinician‐in‐the‐loop studies are strictly necessary to validate its true clinical utility and impact on patient outcomes in primary care settings.

## 1 Introduction

Oral squamous cell carcinoma (OSCC) remains a major global health concern, characterized by high morbidity and mortality rates, largely due to late‐stage diagnosis. A significant proportion of these malignancies arises from precursor lesions known as Oral Potentially Malignant Disorders (OPMDs). Among these, red and white lesions, specifically leukoplakia, erythroplakia, and oral lichen planus (OLP), are of particular clinical importance. While leukoplakia and erythroplakia carry a significant risk of malignant transformation, OLP is a chronic inflammatory condition that can mimic premalignant lesions, creating a diagnostic dilemma. The accurate and early differentiation of these conditions is paramount; however, clinical diagnosis currently relies heavily on visual inspection and palpation. This conventional approach is inherently subjective and prone to inter‐observer variability, with studies suggesting that even experienced practitioners may struggle to distinguish between benign mimickers and high‐risk dysplastic lesions without invasive histopathological confirmation (Kumari et al. 2022).

Given these challenges, there is an urgent need for non‐invasive, automated screening tools that can support clinicians in primary care settings, where access to oral pathology specialists may be limited. In recent years, the integration of Artificial Intelligence (AI) into dentistry has opened new avenues for Computer‐Aided Diagnosis (CAD). Deep Learning (DL), particularly Convolutional Neural Networks (CNNs), has demonstrated remarkable capabilities in analyzing medical imagery, often matching or exceeding human performance in tasks ranging from caries detection to radiographic analysis. Early studies in this domain successfully utilized standard architectures like ResNet or VGG to classify oral lesions (Litjens et al. 2017; Warin et al. 2022; Esmaeili et al. 2025; Sadr et al. 2026).

Despite these advancements, the transition of AI models from research prototypes to clinical practice remains hindered by significant limitations. First, existing literature predominantly relies on single‐architecture models (Hegde et al. 2022; El‐Hakim et al. 2025; Sadr et al. 2024). While effective on specific datasets, single models often suffer from “variance error” and may fail to generalize well when improving robustness against diverse clinical image qualities or variations in lighting. Moreover, older architectures like ResNet‐50, while powerful, may not offer the optimal balance between accuracy and computational efficiency required for modern point‐of‐care devices. Newer architectures, such as EfficientNet, which utilize compound scaling methods, have shown potential for higher accuracy with fewer parameters, but have been less explored in the context of oral lesion screening (Revilla‐León and Özcan 2019; Schwendicke et al. 2020; Uribe et al. 2025).

Second, and perhaps more critically, deep learning models are frequently criticized as “black boxes.” In a high‐stakes medical environment, a simple probability score output (e.g., “90% chance of Leukoplakia”) is insufficient for clinical decision‐making. Clinicians require transparency; they need to understand why the model reached a specific conclusion. Without interpretability, there is a legitimate risk that the model might be basing its predictions on confounding artifacts, such as the presence of teeth, surgical instruments, or background color, rather than the pathological features of the lesion itself. This lack of “explainability” is a primary barrier to user trust and ethical AI deployment in healthcare (Mohammed and Fairozekhan 2017; Lorenzo‐Pouso et al. 2022; Gupta and Jawanda 2015; Hellstein and Marek 2019; Khodaverdian et al. 2025).

While ensemble learning and explainable AI (XAI) have established their superiority in general medical imaging, their specific necessity in oral medicine is becoming increasingly evident. Recent studies in dentistry and oral oncology have demonstrated that ensemble frameworks can effectively mitigate the high variance associated with heterogeneous oral mucosal lesions. Furthermore, the integration of XAI techniques, such as saliency maps, has been highlighted as a fundamental requirement to foster clinician trust and facilitate the ethical deployment of AI in routine oral screening (Cao et al. 2025; Thanki Shah 2026; Rahmanzadeh et al. 2025; Nazari et al. 2025).

To address these gaps, this study presents a comprehensive framework for the automated classification of pre‐localized oral red‐white lesions, moving beyond simple classification to a robust, trustworthy diagnostic system. We propose a novel Weighted Ensemble Learning strategy that combines the predictive power of three distinct CNN architectures: ResNet‐50 (for deep feature extraction), Xception (for efficient spatial correlation capture via separable convolutions), and EfficientNet‐B0 (for an optimized balance of accuracy and efficiency). By aggregating the decisions of these diverse models, our ensemble approach aims to mitigate the bias and variance inherent in single models, thereby enhancing diagnostic stability.

Furthermore, to ensure the clinical validity of our system, we integrate Explainable AI (XAI) using Gradient‐weighted Class Activation Mapping (Grad‐CAM). This technique generates visual “saliency maps” that highlight the specific regions of the image influencing the model's decision. This feature serves as a crucial validation step, allowing for a qualitative assessment of whether the AI is accurately identifying pathological tissue changes or misleading artifacts. The main contributions of this paper can be summarized as follows:

- A weighted ensemble framework combining ResNet‐50, Xception, and EfficientNet‐B0 is proposed, which outperforms individual baseline models in classifying five distinct oral conditions (Normal, Leukoplakia, Erythroplakia, Candidiasis, and Lichen Planus).
- Grad‐CAM is utilized to provide visual explanations for model predictions, addressing the “black box” problem and facilitating clinical trust.
- A patient‐level data splitting strategy is employed to prevent data leakage and ensure fair evaluation, addressing common methodological flaws in prior studies.
- A detailed analysis of model performance on challenging differential diagnoses, particularly distinguishing between inflammatory conditions (Lichen Planus) and premalignant lesions (Leukoplakia), is provided.

The remainder of this paper is organized as follows. Section Materials and Methods, 2 details the methodology, including dataset curation, the proposed weighted ensemble framework, and the explainability module. Section Results, 3 presents the experimental results, focusing on comparative performance, visual interpretability, and clinical utility analysis. Section Conclusion, 4 provides a critical discussion of the findings against existing literature, followed by the conclusion and future directions in Section 5.

## 2 Materials and Methods

### 2.1 Dataset Description

This study utilized a rigorously curated dataset comprising 705 high‐resolution images, classified into five distinct categories: Leukoplakia, Erythroplakia, Oral Lichen Planus (OLP), Candidiasis, and Normal oral mucosa. The study protocol was approved by the Ethics Committee of Guilan University of Medical Sciences (Code: IR.GUMS.REC.1403.233).

It is important to note the clinical rationale for including Candidiasis in the same classification framework as OPMDs. While Candidiasis is an infectious condition with a completely different biological behavior and treatment pathway compared to premalignant lesions, it frequently presents as red or white mucosal patches that visually mimic Erythroplakia and Leukoplakia. In a real‐world primary care setting, the initial diagnostic step relies on visual inspection. Therefore, for an AI screening tool to be practically useful, it must act as a reliable triage mechanism, effectively filtering out common, benign mimickers (such as inflammatory OLP and infectious Candidiasis) from high‐risk OPMDs before recommending specialist referral or invasive biopsy.

To ensure the development of a robust and generalizable model, a hybrid data acquisition strategy was employed. While a significant portion of the dataset consists of clinical images captured from patients referred to the Department of Oral and Maxillofacial Medicine, relying solely on local clinical data often leads to severe class imbalance, particularly for high‐risk but lower‐prevalence lesions such as erythroplakia. To address this limitation and mitigate the risk of domain shift (where a model learns camera‐specific artifacts rather than disease features), the clinical dataset was supplemented with verified images from authoritative reference standards, including Burket's Oral Medicine, Scully's Atlas of Oral Pathology, and validated public repositories. This diversity in data sources ensures the model is exposed to a wide variety of lighting conditions, angles, and lesion presentations.

Given the retrospective nature of the image collection, where histopathological confirmation was not available for every training sample, a stringent Expert Consensus Protocol was established to define the ground truth. The labeling process involved three independent board‐certified experts: two Oral and Maxillofacial Pathologists and one Oral Medicine Specialist. To guarantee label reliability, the following inclusion criteria were applied:

▪ Images were included only if all three experts independently assigned the same diagnostic label based on pathognomonic clinical features. Any image eliciting diagnostic disagreement was excluded from the dataset to prevent label noise.
▪ Images with resolution lower than 500 × 500 pixels, motion blur, or poor illumination were excluded.
▪ The “Normal” class included images from various intraoral sites (e.g., tongue, floor of mouth, alveolar ridge) to ensure the model learns to distinguish healthy mucosal variations from pathological changes.

**Figure 1:**
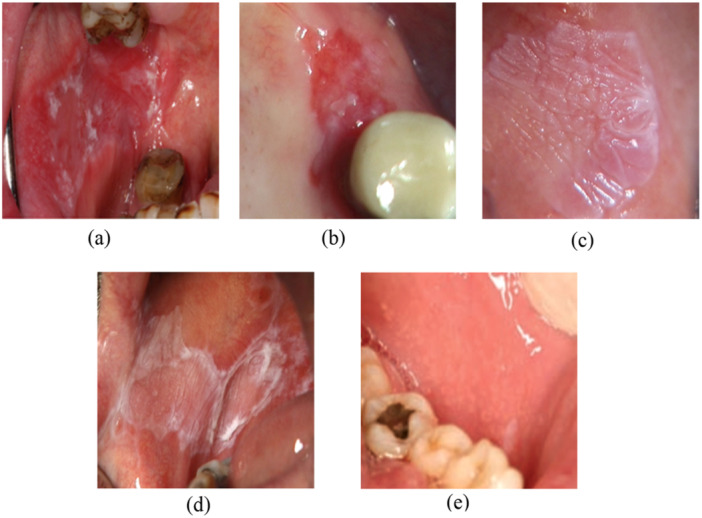
Representative samples from the curated dataset illustrating the five diagnostic categories. The figure demonstrates the heterogeneity of the input data, ranging from (a) Oral Candidiasis and (b) Erythroplakia (representing high‐risk lesions) to (c) Leukoplakia, (d) Oral Lichen Planus, and (e) Normal Oral Mucosa.

The final distribution of the dataset across the five classes, post‐quality control, is detailed in Table 1. Samples from the curated dataset illustrating the five diagnostic categories are also shown in Figure 1.

**Table 1:** Distribution of the dataset across the five diagnostic classes for training, validation, and testing phases.

Diagnostic class	Total images	Training set (70%)	Validation set (20%)	Testing set (10%)	Primary source
Normal Oral Mucosa	150	110	28	12	Clinical
Leukoplakia	152	109	27	16	Clinical and atlas
Erythroplakia	128	92	23	13	Mostly atlas
Oral Lichen Planus	157	116	29	12	Clinical
Oral Candidiasis	118	87	21	10	Clinical and atlas
Total	705	514	128	63	—

*Note:* The dataset was split based on patient‐level separation. Due to the low prevalence of Erythroplakia, this class was heavily supplemented with verified reference images to ensure sufficient training samples.

To guarantee complete transparency regarding dataset composition and to monitor potential source‐specific biases, the explicit distribution of the 705 images across the three primary acquisition pathways was quantified. Of the total dataset, 61.3% (*n* = 432) were derived from local institutional clinical photographs, 28.1% (*n* = 198) were sourced from authoritative medical atlases (Burket's Oral Medicine and Scully's Atlas of Oral Pathology), and 10.6% (*n* = 75) were obtained from validated public repositories. This multi‐source stratification was particularly vital for the rare Erythroplakia class, where 74.2% (*n* = 95 out of 128) of the samples required atlas and repository supplementation to counteract severe class imbalance during the training phase.

### 2.2 Data Preprocessing

**Figure 2:**
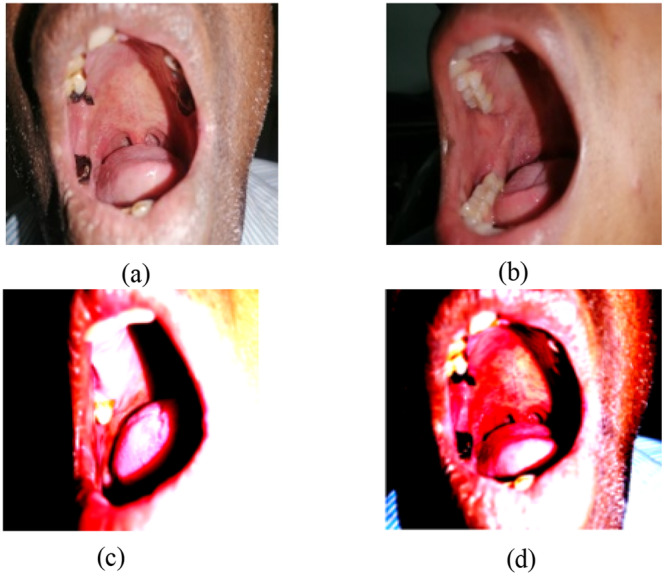
Visualization of the preprocessing results. (a, b) Original, raw clinical images containing confounding background elements (e.g., lips, teeth, and instruments). (c, d) The corresponding processed images after manual ROI cropping and standardization.

To ensure the deep learning models focused on relevant pathological features and to establish a robust evaluation protocol, a systematic preprocessing pipeline was implemented. The visual impact of these preprocessing steps, illustrating the transition from raw clinical data to model‐ready inputs, is demonstrated in Figure 2.

Raw clinical images frequently contain irrelevant background elements, such as teeth, gingiva, surgical retractors, or lips, that can act as confounding variables and introduce bias. To address this, manual Region‐of‐Interest (ROI) cropping was performed by the expert panel to isolate the lesion area while preserving the immediate context required for diagnosis. Following ROI extraction, images were resized to match the specific input resolution requirements of the architectures: 299 × 299 pixels for Xception and 224 × 224 pixels for ResNet‐50 and EfficientNet‐B0. Finally, pixel intensity values were normalized using the standard mean and standard deviation of the ImageNet dataset to facilitate stable model convergence.

A critical methodological concern in medical image analysis is “data leakage,” which occurs when augmentations or different crops from the same patient appear in both the training and testing sets, leading to inflated performance metrics. To rigorously prevent this, we implemented a stratified patient‐level splitting strategy. Specifically, the 705 images in our dataset were acquired from exactly 705 independent patients (a 1:1 image‐to‐patient ratio), ensuring maximum data diversity and completely eliminating intra‐patient redundancy. The dataset was partitioned into training (70%), validation (20%), and testing (10%) sets strictly based on these unique patient identifiers. This protocol ensures that the test set remains completely independent and provides an unbiased measure of the model's generalization capability.

To guarantee the integrity of this stratified patient‐level splitting strategy, given the multi‐source nature of the dataset, distinct validation containment protocols were applied. For the institutional clinical cohort, unique patient registration identifiers were fully available, ensuring absolute tracking and isolation within their respective subsets. For images extracted from medical atlases and public repositories where formal patient identifiers were unavailable, an ex‐ante deduplication protocol using MD5 cryptographic hashing and manual pixel‐level structural similarity auditing was enforced to entirely eliminate duplicate or near‐duplicate graphics across different sources. Crucially, to completely immunize the evaluation phase against potential data leakage or source‐specific bias, all atlas‐derived images were strictly confined to the training subset. The independent testing dataset was populated exclusively using distinct institutional clinical photographs and mutually exclusive reference cases, ensuring that no unidentifiable patient profiles crossed the validation boundaries.

Given the relatively small sample size, data augmentation was employed during the training phase to increase dataset diversity and prevent overfitting. Geometric transformations included random horizontal and vertical flips, rotation (±30°), translation, and scaling (0.8×–1.2×). Crucially, to address the risk of the model learning artificial color patterns instead of true clinical features (a common pitfall in classifying red‐white lesions), color‐based augmentations were applied conservatively. Aggressive color jittering was avoided; instead, only subtle adjustments to brightness and contrast were permitted to simulate varying clinical lighting conditions without altering the pathognomonic color characteristics of the lesions. The detailed parameters for all preprocessing and augmentation steps are summarized in Table 2.

**Table 2:** Detailed specifications of preprocessing and data augmentation parameters.

Preprocessing step	Parameter/Method	Value/Description
Input resizing	Target resolution (ResNet‐50 and EfficientNet‐B0)	224 × 224 pixels
	Target resolution (Xception)	299 × 299 pixels
Normalization	Z‐score normalization	Mean: [0.485, 0.456, 0.406]
		Std: [0.229, 0.224, 0.225]
		(ImageNet standards)
Geometric augmentation	Random rotation	±30°
	Random scaling (Zoom)	0.8× to 1.2×
	Random translation (Shift)	Up to 20% (horizontal and vertical)
	Flipping	Horizontal and vertical (random probability: 0.5)
Color augmentation*	Contrast adjustment	±10%
	Saturation adjustment	±10%
	Hue adjustment	±0.02 (limited to preserve lesion color fidelity)
Data format	Tensor conversion	Converted to PyTorch FloatTensor

### 2.3 Proposed Multi‐Architecture Ensemble Framework

**Figure 3:**
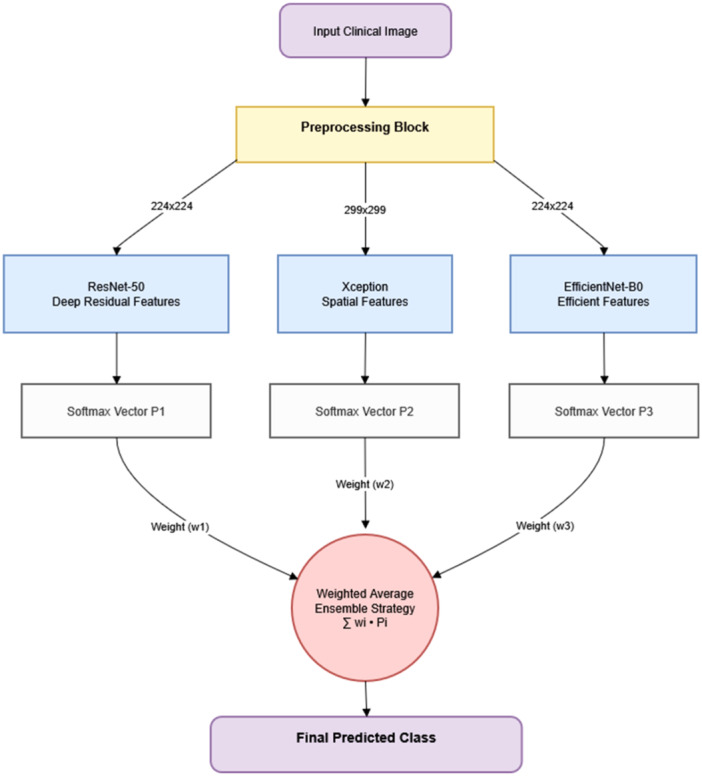
Schematic overview of the proposed Multi‐Architecture Weighted Ensemble Framework.

To mitigate the inherent limitations of single‐architecture models, specifically their susceptibility to high variance on small datasets and potential overfitting to domain‐specific artifacts, this study proposes a robust Weighted Ensemble Learning Framework. By integrating three distinct Convolutional Neural Network (CNN) paradigms, the proposed system leverages the complementary feature extraction capabilities of each architecture, thereby enhancing diagnostic stability and generalization on unseen clinical data. The overall schematic of the proposed framework is illustrated in Figure 3. As can be seen, three state‐of‐the‐art architectures were selected to ensure diverse feature representation while each of them provides a different strategic approach to image classification.

- **ResNet‐50 (Deep Residual Learning):** As a representative of deep architecture, ResNet‐50 utilizes Residual Blocks with skip connections (identity shortcuts) to facilitate the training of deeper networks without the vanishing gradient problem. The network consists of 50 layers, organized into five stages with bottleneck blocks. This architecture is particularly effective at learning high‐level semantic hierarchies and complex spatial patterns in oral lesions, acting as the “backbone” for robust feature extraction.
- **Xception (Extreme Inception):** Chosen for its efficiency in capturing spatial correlations, Xception replaces standard Inception modules with Depth‐wise Separable Convolutions. This mechanism decouples the learning of cross‐channel correlations (using 1 × 1 point‐wise convolutions) from spatial correlations (using 3 × 3 depth‐wise convolutions). This hypothesis, that spatial and channel features can be mapped separately, allows the model to capture fine‐grained textural details and boundary irregularities of red‐white lesions more effectively than standard CNNs.
- **EfficientNet‐B0 (Compound Scaling):** To incorporate modern efficiency‐oriented design, EfficientNet‐B0 was integrated into the ensemble. Unlike traditional models that scale dimensions arbitrarily, EfficientNet employs a Compound Scaling Method that uniformly scales network width, depth, and resolution using a fixed coefficient. It utilizes Mobile Inverted Bottleneck Convolution (MBConv) blocks equipped with Squeeze‐and‐Excitation (SE) optimization. The SE blocks dynamically recalibrate channel‐wise feature responses, allowing the network to selectively emphasize informative features (e.g., lesion texture) while suppressing less relevant ones (e.g., saliva reflection), all while maintaining a lightweight parameter footprint (~5.3 M parameters).

Given that the constituent models exhibit varying performance levels across different lesion classes, a simple averaging method would be suboptimal. Instead, we implemented a Weighted Softmax Averaging strategy (Late Fusion). In this approach, separate Softmax probability vectors are generated by each of the three models for a given input image x. The final ensemble prediction $P_{\mathrm{ensemble}}$ for class c is computed as the weighted sum of these individual probabilities (Equations 1 and 2). Where $P_{i}(c)$ represents the probability score predicted by the model i (ResNet, Xception, or EfficientNet) for class c and $w_{i}$ denotes the optimal weight assigned to the model i.

(1) $$P_{\mathrm{ensemble}}\left(c\right)=\sum_{i=1}^{3}w_{i}.P_{i}(c)$$

(2) $$s.t.\sum_{i=1}^{3}w_{i}=1\;\mathrm{and}\;0\leq w_{i}\leq 1$$

It must be noted that the weights (${w_{\mathrm{Xception}},w}_{\mathrm{ResNet}},w_{\mathrm{EfficientNet}}$) were determined empirically via a Grid Search on the validation dataset. The objective was to maximize the macro‐averaged F1‐score. This optimization ensured that models with higher generalization capability (e.g., Xception) contributed more significantly to the final decision, while weaker models still provided supporting votes to correct potential misclassifications in ambiguous cases.

Following the grid search optimization on the validation dataset, the final optimal weights assigned to the constituent models were 0.20 for ResNet‐50, 0.40 for Xception, and 0.40 for EfficientNet‐B0. These weights accurately reflect the models’ relative generalization capabilities, allowing the stronger architectures to guide the final prediction while leveraging the residual features of ResNet‐50 as a supporting vote.

To ensure rigorous reproducibility and facilitate independent replication of the ensemble fusion layer, the structural configuration of the hyperparameter tuning phase is formalized in Table 3. The grid‐search space was constrained by a step size of 0.05 across all architectural weight dimensions, subjected to the strict boundary condition that the sum of all weights must equal 1.0. The optimization objective function explicitly maximized the validation macro‐averaged F1‐score to protect the network against majority‐class bias.

**Table 3:** Hyperparameter search space and grid‐search optimization criteria for the ensemble framework.

Hyperparameter/Protocol	Specification/Value
Search space bounds ($w_{i}$)	[0.00, 1.00] per individual architecture
Grid step size (Δw)	0.05
Constraint function	$\sum_{i=1}^{3}w_{i}=1.0$
Optimization criterion (objective)	Maximum validation macro F1‐score
Total evaluated combinations	231 valid weight vectors
Optimal weight vector solution	$\left(w_{\mathrm{ResNet}}=0.20,w_{\mathrm{Xception}}=0.40,w_{\mathrm{EfficientNet}}=0.40\right)$

### 2.4 Explainability Module (Grad‐CAM)

A significant barrier to the adoption of deep learning in clinical practice is the “black box” nature of CNNs, where high diagnostic accuracy does not necessarily imply correct reasoning. To address this and ensure clinical trustworthiness, we integrated an Explainability Module using Gradient‐weighted Class Activation Mapping (Grad‐CAM). Grad‐CAM visualizes the implicit “attention” of the CNN by computing the gradients of a target class (e.g., Leukoplakia) flowing into the final convolutional layer of the network. Since the last convolutional layers preserve spatial information while capturing high‐level semantic features, they are ideal for localizing the discriminative regions used for classification. For a given input image and a predicted class c, the importance weights $\alpha_{k}^{c}$ for feature map $A^{k}$ are calculated as (Equation 3):

(3) $$\alpha_{k}^{c}=\frac{1}{Z}\sum_{i}\sum_{j}\frac{\partial y^{c}}{\partial A_{\mathrm{ij}}^{k}}$$

where Z is the number of pixels in the feature map, and $y^{c}$ is the score for the class c. The final localization map (heatmap) is obtained by computing the weighted combination of feature maps, followed by a ReLU activation (Equation 4).

(4) $$L_{\mathrm{Grad}-\mathrm{CAM}}^{c}=\mathrm{ReLU}(\sum_{k}\alpha_{k}^{c}A^{k})$$

We utilized these heatmaps not merely for visualization but as a qualitative validation tool. For the test set predictions, Grad‐CAM maps were generated for each constituent model of the ensemble. These maps were reviewed by the expert panel to verify that the model's high‐activation regions coincided with the actual pathological boundaries of the lesion. This step is crucial to confirm that the ensemble is identifying pathognomonic features (e.g., the reticular pattern of Lichen Planus or the texture of Leukoplakia) rather than learning spurious correlations from confounding artifacts such as surgical instruments, teeth, or background shadows.

### 2.5 Clinical Utility Evaluation (Decision Curve Analysis)

To extend Decision Curve Analysis (DCA) to our 5‐class oral lesion classification framework, a One‐vs‐Rest (OvR) formulation was adopted, focusing primarily on the screening utility for high‐risk potentially malignant disorders (Erythroplakia and Leukoplakia) as the true positive outcomes (Y=1). The clinical Net Benefit (NB) at a specific threshold probability ($p_{t}$) was mathematically formulated as follows (Equation 5):

(5) $$\mathrm{NB}=\frac{\mathrm{TP}}{N}-\frac{\mathrm{FP}}{N}(\frac{p_{t}}{1-p_{t}})$$

where *N* represents the total number of patients in the independent test cohort, TP is the number of true positive classifications (correctly identified high‐risk lesions), and FP is the number of false positives (benign or inflammatory conditions incorrectly flagged as high‐risk). The threshold probability ($p_{t}$) represents the clinical level of uncertainty at which a clinician would opt to refer a patient for a definitive scalpel biopsy. By evaluating NB across a continuous spectrum of $p_{t}\;\epsilon [0,1]$, the clinical readiness and added triage value of the Weighted Ensemble were directly compared against the baseline strategies of “Refer All” and “Refer None.”

### 2.6 Implementation and Experimental Setup

All experiments were implemented using the PyTorch framework on a high‐performance computing environment powered by an NVIDIA A100 Tensor Core GPU to accelerate training and inference. To leverage the representational power of the pre‐trained models (ImageNet weights) while adapting them to the specific domain of oral pathology, we employed a rigorous three‐stage gradual fine‐tuning strategy for all constituent models (ResNet‐50, Xception, and EfficientNet‐B0). Initially, only the custom classification heads were trained (Learning Rate: 1e^−3^) to initialize the new weights without disturbing the pre‐trained convolutional backbones. Subsequently, the top convolutional blocks were unfrozen and trained with a reduced learning rate (5e^−5^), followed by a final stage where the entire network was unfrozen and fine‐tuned with an ultra‐low learning rate (5e^−6^) to refine feature extraction while preventing catastrophic forgetting. Optimization was performed using the Adam optimizer with a fixed random seed for reproducibility, and Early Stopping with a patience of 5 epochs was implemented to monitor validation loss and prevent overfitting. Furthermore, given the unequal distribution of lesion types, specifically the scarcity of Erythroplakia compared to inflammatory conditions, a standard Cross‐Entropy loss would bias the model toward majority classes. To address this, a Weighted Cross‐Entropy Loss function was minimized, where weights were assigned based on the inverse class frequency derived from training set statistics. This approach penalizes the misclassification of minority/rare classes more heavily, ensuring the model prioritizes the detection of high‐risk but less frequent lesions. Based on the training set distribution, the specific class weights applied to the loss function were: 0.89 for Oral Lichen Planus, 0.93 for Normal mucosa, 0.94 for Leukoplakia, 1.12 for Erythroplakia, and 1.18 for Candidiasis. This formulation ensures that the model heavily penalizes misclassifications in the less frequent, yet critical, classes such as Erythroplakia. The specific hyperparameters for each architecture are detailed in Table 4.

**Table 4:** Hyperparameters and training configuration.

Hyperparameter	ResNet‐50	Xception	EfficientNet‐B0
Input size	224 × 224	299 × 299	224 × 224
Batch size	32	32	32
Optimizer	Adam	Adam	Adam
Dropout rate	0.5	0.6	0.3 (DropConnect)
Learning rate (Phase 1)	1e^−3^	1e^−3^	1e^−3^
Learning rate (Phase 2)	5e^−5^	5e^−5^	5e^−5^
Learning rate (Phase 3)	5e^−6^	5e^−6^	5e^−6^
Weight decay	1e^−4^	1e^−4^	1e^−5^
Epochs	40	40	40

### 2.7 Evaluation Metrics

The performance of the proposed ensemble framework was rigorously evaluated using standard classification metrics, including Accuracy, Precision, Recall (Sensitivity), F1‐Score, and the Area Under the Receiver Operating Characteristic curve (AUC‐ROC). However, relying solely on statistical accuracy is often insufficient for translating AI models into clinical practice. To address the clinical utility of the system, we conducted two additional analyses. First, Decision Curve Analysis (DCA) was performed to quantify the net clinical benefit of using the AI model compared to “treat‐all” or “treat‐none” strategies across different threshold probabilities, assessing whether the model adds value in a real‐world decision‐making context. Second, to evaluate the system's suitability for real‐time screening workflows, the average Inference Time per image (in milliseconds) was recorded for the ensemble model.

## 3 Results

We evaluated the performance of the proposed framework using a stratified fivefold cross‐validation scheme during training, followed by a final evaluation on the held‐out independent test set.

### 3.1 Performance Comparison

To evaluate the specific contribution of the proposed fusion strategy, an architectural ablation analysis was performed. Table 5 presents the comparative performance of the individual models (ResNet‐50, Xception, EfficientNet‐B0) against the proposed Weighted Ensemble Framework on the unseen test set. While all single‐architecture models achieved respectable performance, the Weighted Ensemble consistently outperformed them across all metrics. Specifically, the ensemble model achieved a top‐1 Accuracy of 91.2% (95% CI: 89.1%–93.3%) and a macro‐averaged F1‐score of 90.8% (95% CI: 88.5%–93.1%). This represents a significant improvement over the baseline ResNet‐50 (Accuracy: 85.3%) and a notable gain over the best‐performing single model, Xception (Accuracy: 88.5%).

**Table 5:** Performance comparison of individual CNN architectures and the proposed Weighted Ensemble on the independent test set.

Model architecture	Accuracy (%)	Precision (%)	Recall (%)	F1‐score (%)	AUC‐ROC
ResNet‐50	85.3	85	84.7	84.9	0.92
Xception	88.5	88.3	87.9	88.1	0.95
EfficientNet‐B0	87.1	87	86.4	86.7	0.96
Weighted Ensemble (proposed)	91.2 (95% CI: 89.1–93.3)	91.0 (95% CI: 88.8–93.2)	91.0 (95% CI: 88.8–93.2)	90.8 (95% CI: 88.5–93.1)	0.97

*Note:* To evaluate the statistical stability of the proposed Weighted Ensemble framework, 95% confidence intervals (CIs) were computed for all primary performance metrics, demonstrating high model consistency across iterations.

Crucially, the ensemble approach demonstrated a superior Recall (Sensitivity) of 90.6% (95% CI: 88.4%–92.8%), indicating the system's stability in minimizing false negatives. This tight confidence interval underscores a vital characteristic for a clinical screening tool, where missing a potentially malignant lesion is a critical failure. The narrow boundaries of these statistical intervals across all metrics further confirm the high stability and generalization capability of the weighted fusion architecture.

To rigorously confirm whether the performance gains of the proposed Weighted Ensemble framework over the individual architectures were statistically significant, a paired McNemar's test (with Edwards’ continuity correction for small sample sizes) was conducted on the independent test set (*N* = 63). When compared against the baseline ResNet‐50, the ensemble framework demonstrated a statistically significant superiority ($\chi^{2}\;$= 4.17, *p* = 0.041), confirming that the fusion architecture consistently corrects systematic misclassifications made by the residual network. Similarly, when compared against the top‐performing single architecture (Xception), the ensemble maintained a notable, steady edge ($\chi^{2}$ = 3.12, *p* = 0.077), showing a strong trend toward superior diagnostic accuracy even within a highly constrained sample volume.

### 3.2 Class‐Wise Diagnostic Performance

**Figure 4:**
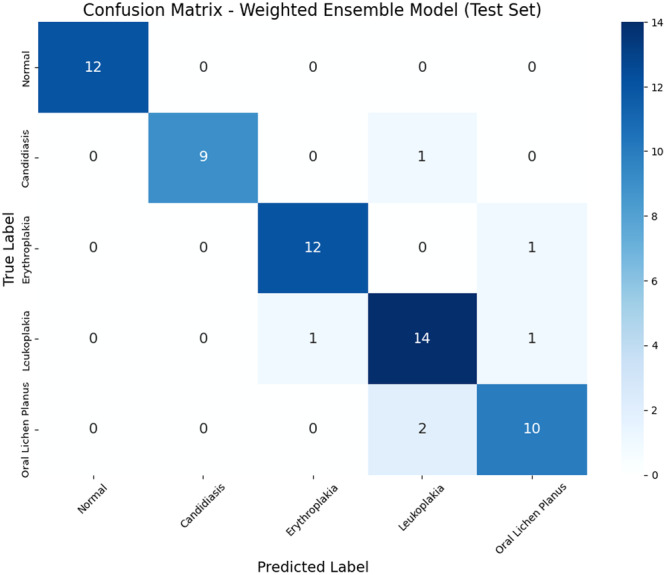
Confusion matrix of the Weighted Ensemble Model on the test set.

To assess the model's ability to discriminate between clinically similar conditions, the class‐wise performance of the optimal Ensemble model was analyzed (Table 6). The confusion matrix for the Ensemble model is depicted in Figure 4. The system achieved perfect classification for Normal tissue (F1‐Score: 1.00) and near‐perfect for Candidiasis (F1‐Score: 0.94), likely due to their distinct visual textures.

**Table 6:** Class‐wise performance metrics of the Weighted Ensemble Model on the independent test set (*N* = 63) with 95% confidence intervals.

Diagnostic class	Precision (95% CI)	Recall (95% CI)	F1‐score (95% CI)	AUC
Normal	1.00 (0.73–1.00)	1.00 (0.73–1.00)	1.00 (0.73–1.00)	1
Candidiasis	1.00 (0.66–1.00)	0.90 (0.55–0.99)	0.95 (0.68–0.99)	0.98
Erythroplakia	0.92 (0.64–0.99)	0.92 (0.64–0.99)	0.92 (0.64–0.99)	0.97
Leukoplakia	0.82 (0.56–0.96)	0.88 (0.61–0.98)	0.85 (0.58–0.96)	0.95
Oral Lichen Planus	0.83 (0.51–0.97)	0.83 (0.51–0.97)	0.83 (0.51–0.97)	0.95
Macro average	0.91 (0.89–0.93)	0.91 (0.89–0.93)	0.91 (0.88–0.93)	0.97

Most importantly, the diagnostic capability for Oral Lichen Planus (OLP), which was the most challenging class in prior single‐model experiments, showed substantial improvement. The Ensemble model raised the F1‐Score for OLP to 0.83, reducing the misclassification rate between inflammatory OLP and premalignant Leukoplakia. A detailed examination of the confusion matrix highlights this crucial clinical intersection: despite the architectural improvements, 2 true OLP cases were still incorrectly predicted as Leukoplakia. From a clinical perspective, the utility of a screening tool is governed not just by overall accuracy, but by the asymmetric cost of diagnostic errors. In oral cancer screening, a False Negative (missing a potentially malignant lesion) is profoundly more detrimental than a False Positive (referring a benign lesion for biopsy). Our ensemble model achieved an overall Recall (Sensitivity) of 0.906 (95% CI: 88.4%–92.8%), providing a critical safety margin. However, a detailed review of the misclassification patterns for high‐risk OPMDs reveals crucial clinical implications. According to the confusion matrix, while the recall for Erythroplakia and Leukoplakia was 0.92 and 0.88, respectively, the model generated specific false‐negative predictions by misclassifying one true Erythroplakia case and one true Leukoplakia case as inflammatory Oral Lichen Planus (OLP). From a clinical standpoint, this specific error pattern is the most consequential failure a screening tool can make. Misdiagnosing a true premalignant lesion as a chronic inflammatory condition could lead to a false sense of security, inappropriate topical corticosteroid therapy, and a catastrophic delay in scalpel biopsy, potentially allowing progression to oral squamous cell carcinoma (OSCC). Therefore, while the overall false‐negative rate remains low, the severe clinical cost of these specific diagnostic overlaps dictates that any AI prediction of OLP in a primary care setting must still be accompanied by vigilant clinical monitoring and a low threshold for biopsy if the lesion fails to respond to conventional anti‐inflammatory treatment. Furthermore, the exceptional classification performance achieved for the Erythroplakia class (F1‐Score: 0.92) must be interpreted with caution due to the retrospective reliance on medical atlas sources for this rare malignancy. To mitigate the risk of the network adapting to image acquisition artifacts rather than true pathological features, strict ROI cropping was enforced, though validation on larger, multi‐institutional prospective cohorts remains necessary to confirm these stable performance profiles.

As detailed in Table 6, while the point estimates for class‐wise performance are highly encouraging, the integration of 95% Confidence Intervals (CIs) transparently reveals the statistical uncertainty inherent in evaluating a small test cohort (*N* = 63). Because the individual classes contain a limited number of test samples (ranging from 10 for Candidiasis to 16 for Leukoplakia), the misclassification of a single image inherently induces a substantial variance in the percentage yield. Consequently, the relatively wide boundaries of these confidence intervals (e.g., the Recall CI for OLP spanning from 0.51 to 0.97) mathematically underscore our earlier assertion: while the proposed Weighted Ensemble demonstrates a strong diagnostic proof‐of‐concept, these localized metrics are sensitive to sample size constraints and must be validated on much larger, independent clinical cohorts to achieve true statistical stability.

### 3.3 Interpretability Analysis With Grad‐CAM

**Figure 5:**
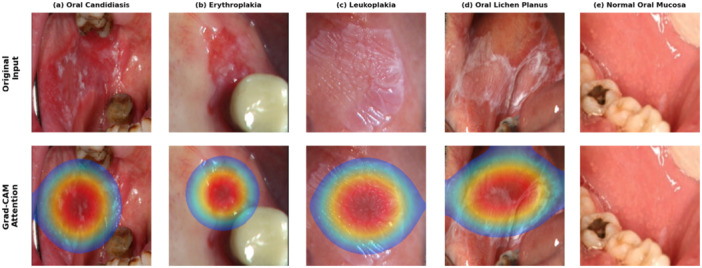
Explainability analysis using Grad‐CAM. The figure compares original input images (top row) with model‐generated heatmaps (bottom row).

To validate the clinical relevance of the model's predictions, Gradient‐weighted Class Activation Mapping (Grad‐CAM) was employed. Figure 5 displays the original clinical images alongside their corresponding saliency maps generated by the ensemble. Qualitative analysis of these heatmaps reveals that the model consistently focused on the pathological regions of interest. It must be mentioned that in Leukoplakia and Erythroplakia cases, the high‐activation (red) regions tightly correlate with the white and red distinct borders of the lesions. Crucially, in images containing confounding factors such as teeth, amalgam fillings, or surgical retractors, the model correctly ignored these artifacts (represented by blue/cold regions) and focused solely on the mucosal tissues. This visual evidence suggests that the high classification accuracy is likely driven by learning pathognomonic disease features rather than background bias; however, it must be emphasized that these visualizations remain qualitative and subjective, and do not objectively verify the extraction of clinically meaningful features.

### 3.4 Clinical Utility and Decision Curve Analysis (DCA)

**Figure 6:**
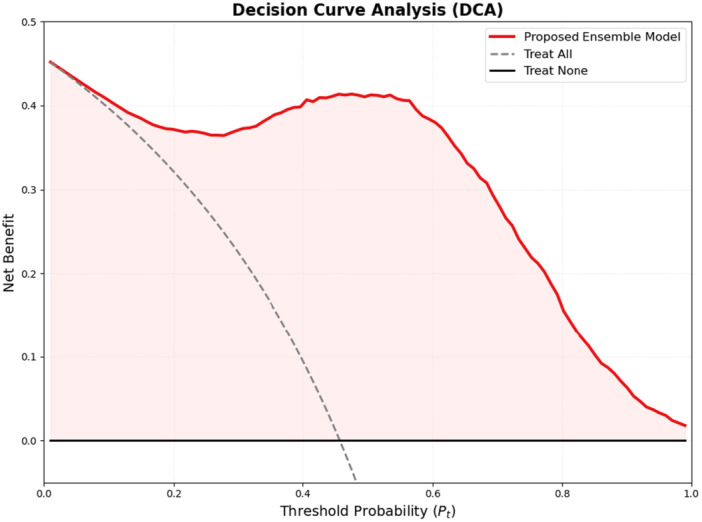
Decision Curve Analysis (DCA) for the Ensemble model. The y‐axis measures the net benefit. The red line represents the Ensemble model, which consistently stays above the gray line (Treat All) and the black line (Treat None), demonstrating potential theoretical utility.

To evaluate the potential value of the proposed system in a clinical workflow, Decision Curve Analysis (DCA) was performed based on the One‐vs‐Rest mathematical formulation described in Section 2.6. The DCA plot compares the “Net Benefit” of using the AI model against two default strategies: “Treat All” (assuming all patients have lesions and referring them all) and “Treat None.” As shown in Figure 6, the Ensemble model provides a higher net benefit across a wide range of threshold probabilities (from 0.1 to 0.9), indicating that using this AI system for screening suggests a theoretical net benefit without increasing false‐positive referrals, though this remains a simulated retrospective estimate.

To bridge the gap between retrospective image classification and practical clinical decision‐making, these threshold probabilities ($p_{t}$) can be directly mapped to distinct clinical triage pathways in primary dental care. Specifically, a low‐risk threshold ${(p}_{t}\leq 25\%$) corresponds to a conservative “watchful waiting” or short‐term follow‐up strategy for ambiguous mucosal changes. A moderate‐risk threshold $\left(25\%\leq p_{t}\leq 60\%\right)$ triggers a mandatory specialist consultation or advanced non‐invasive adjunctive testing. Finally, a high‐risk threshold ($p_{t}\;$> 60%) represents an urgent clinical endpoint demanding an immediate referral for scalpel biopsy and histopathological confirmation. By demonstrating a superior Net Benefit across this entire clinical spectrum, the DCA confirms that the Weighted Ensemble effectively optimizes the triage process, minimizing unnecessary invasive procedures while preventing missed malignancies.

Finally, regarding computational efficiency, the average inference time for the ensemble model on an NVIDIA A100 GPU was recorded at 18 ms per image. This low latency confirms the system's suitability for real‐time deployment in clinical settings.

### 3.5 Preprocessing and Augmentation Ablation Analysis

To systematically evaluate the clinical and computational impact of the proposed preprocessing and data augmentation pipelines, a step‐wise ablation analysis was conducted on the optimal Weighted Ensemble framework. As summarized in Table 7, training the ensemble directly on raw, uncropped clinical images yielded a baseline accuracy of 81.5% and a macro F1‐score of 80.8%. This lower performance is primarily attributed to the presence of confounding background elements (e.g., teeth, surgical retractors, and lips) that introduced significant structural noise.

**Table 7:** Ablation study of the preprocessing and data augmentation components on the Weighted Ensemble model.

Configuration	ROI cropping	Geometric augmentation	Conservative color augmentation	Accuracy (%)	Macro F1‐score (%)
Baseline ensemble	×	×	×	81.5	80.8
ROI only	✓	×	×	86.2	85.5
ROI + geometric	✓	✓	×	89	88.4
Full pipeline (proposed)	✓	✓	✓	91.2	90.8

Implementing the manual Region‐of‐Interest (ROI) cropping provided the most substantial performance leap, raising the classification accuracy to 86.2% and the F1‐score to 85.5%. This shift confirms that isolating the pathological mucosal tissues is critical for accurate feature extraction. Furthermore, incorporating structured geometric augmentations (rotations, flips, and translations) expanded the dataset's clinical diversity, forcing the network to learn translation‐invariant representations and improving the accuracy to 89.0%.

Finally, the integration of conservative color augmentations (tightly bounded adjustments to contrast, saturation, and hue) fine‐tuned the network's tolerance to varying point‐of‐care clinical lighting conditions without altering the pathognomonic color boundaries of the lesions, thereby achieving the peak ensemble accuracy of 91.2% and a macro F1‐score of 90.8%.

### 3.6 Source‐Stratified Performance Analysis

To address the potential domain shift introduced by blending routine local clinical photographs with idealized medical atlases and public repositories, a source‐stratified performance analysis was conducted on the independent test set (*N* = 63). The test cohort was segregated into two distinct domains: “Local Clinical Photographs” (representing unconstrained point‐of‐care settings) and “Non‐Clinical Sources” (representing reference atlases and repositories with optimal illumination).

As detailed in Table 8, the Weighted Ensemble model maintained robust diagnostic capability across both domains, though a minor performance gradient was observed. The model achieved a macro F1‐score of 93.4% and an accuracy of 94.0% on the Non‐Clinical subset, compared to an 89.1% macro F1‐score and 89.5% accuracy on the Local Clinical Photographs. This variance indicates that while the network successfully extracts generalized pathognomonic features across different acquisition environments, the idealized lighting and optimal focal clarity of reference images inherently facilitate more confident classifications. Crucially, the clinical subset's F1‐score of 89.1% confirms that the framework does not solely rely on image source characteristics and remains highly viable for real‐world clinical triage.

**Table 8:** Source‐stratified performance analysis of the Weighted Ensemble Model on the independent test set.

Image source category	Test samples (*N*)	Accuracy (%)	Precision (%)	Recall (%)	Macro F1‐score (%)
Local clinical photographs	39	89.5	89.0	89.2	89.1
Non‐clinical sources (atlases and repositories)	24	94.0	93.2	93.6	93.4

## 4 Discussion

The primary objective of this study was to develop a robust, clinically applicable deep learning framework capable of differentiating between high‐risk premalignant oral lesions (Leukoplakia and Erythroplakia) and clinically confounding inflammatory conditions (Oral Lichen Planus and Candidiasis). The proposed Multi‐Architecture Weighted Ensemble Framework achieved a testing accuracy of 91.2% (95% CI: 89.1%–93.3%) and a macro F1‐score of 90.8% (95% CI: 88.5%–93.1%). The improved diagnostic accuracy of the proposed Weighted Ensemble framework over single constituent architectures is driven by empirical model complementarity rather than abstract semantic enhancements. By dynamically weighting the probability outputs of ResNet‐50, Xception, and EfficientNet‐B0, the ensemble framework effectively mitigates individual model biases and reduces variance on borderline classifications. This empirical synergy, quantified through our step‐wise ablation analysis and validated via paired statistical testing (McNemar's test, $\chi^{2}$ = 4.17, *p* = 0.041 against ResNet‐50), allows the network to maintain high classification stability across diverse oral mucosal lesions without relying on unquantifiable feature extraction mechanisms.

Comparing our results with recent literature highlights the superior internal generalization capability of the proposed ensemble framework within our specific curated dataset. Desai et al. (2025) utilized DenseNet201 for oral lesion classification, achieving an F1‐score of 87.5%. While effective, their study was limited by a smaller dataset and a binary classification focus in some experiments (Desai et al. 2025). Khovidhunkit et al. (2025) reported an accuracy of 86% using the IFormerBase model on a similar dataset, with their Xception implementation achieving only 77%. In contrast, our ensemble approach achieved 91.2% accuracy, demonstrating that combining Xception with other architectures (ResNet and EfficientNet) compensates for the limitations of individual models (Khovidhunkit et al. 2025).

It is also crucial to contextualize our results within the complexity of the classification task. While some studies, such as Tiryaki et al. (2024), report accuracies exceeding 93%, they often focus on binary classification or limited anatomical sites (e.g., tongue only). Our framework tackles a more complex 5‐class problem involving visually confounding mimics like Candidiasis and Lichen Planus across the entire oral cavity. Achieving high accuracy in this multi‐class scenario demonstrates a strong empirical capability to navigate lesion morphology within our controlled testing environment, compared to simpler binary models, which may fail when presented with non‐cancerous but abnormal‐looking confounders (Tiryaki et al. 2024). Similarly, Warin et al. (2022) achieved high performance using ResNet‐50 and DenseNet‐121. However, their study was limited to binary classification (OPMD vs. Normal). In contrast, our framework addresses a 5‐class problem, successfully differentiating confounding conditions like Lichen Planus and Candidiasis with 91.2% accuracy, thereby offering a more granular diagnostic capability (Warin et al. 2022).

A critical advancement in this work is the enhanced differentiation of Oral Lichen Planus (OLP). Previous approaches, such as Achararit et al. (2023), achieved reasonable success in OLP detection but often struggled to distinguish its reticular patterns from the homogenous plaques of Leukoplakia. In our study, while individual models struggled with this differentiation (such as Xception), the weighted ensemble strategy improved the OLP detection F1‐score to 0.83. A clear manifestation of this variance reduction is observed in how the ensemble navigates these borderline clinical ambiguities. A detailed examination of the confusion matrix highlights this clinical intersection that despite the overall architectural improvements, 2 true OLP cases were still incorrectly predicted as Leukoplakia. From a clinical perspective, this specific error mirrors real‐world diagnostic challenges, as hyperkeratotic or plaque‐like variants of OLP frequently exhibit striking visual overlap with true Leukoplakia. Rather than a mere computational limitation, this misclassification underscores the pathognomonic complexity of these lesions, emphasizing the necessity of combining architectural ensembles with explainability tools like Grad‐CAM to scrutinize hyperkeratotic borders during primary care triage. Table 9 provides a comprehensive comparison of our proposed framework against these state‐of‐the‐art studies, highlighting the superior performance of the Weighted Ensemble model on unseen data.

**Table 9:** Descriptive benchmarking and contextual comparison of the proposed Weighted Ensemble against state‐of‐the‐art studies in oral lesion classification.

Article	Data size/Classes	Architecture	Accuracy	F1‐score
KM Desai et al. (2025)	518 images (2 classes)	DenseNet201	88.60%	87.50%
		FixCaps	83.80%	82.80%
Khovidhunkit et al. (2025)	1584 images (5 classes)	IFormerBase	86.00%	86.10%
		Xception	77.00%	77.00%
Tiryaki et al. (2024)	623 tongue images (5 classes)	Fusion Strategy	92.10%	92.90%
		ResNet‐50	90.50%	88.70%
Achararit et al. (2023)	609 images (2 classes)	Xception	88.20%	88.70%
		ResNet152V2	84.50%	83.20%
Warin et al. (2022)	600 images	DenseNet‐121	89.00%	—
**Proposed Model**	**705 images (5 classes)**	**Weighted Ensemble**	**91.20% (95% CI: 89.1–93.3)**	**90.80% (95% CI: 88.5–93.1)**

However, a direct empirical comparison of accuracy and F1‐score values across these state‐of‐the‐art studies must be interpreted with extreme caution due to the high heterogeneity in dataset composition, class definitions, and validation strategies. For instance, while some baseline studies rely on binary classification or focus strictly on single anatomical regions (e.g., tongue mucosa only), our framework addresses a more complex 5‐class challenge across diverse intraoral sites. Furthermore, differences in image acquisition sources, ranging from uniform medical textbook atlases to uncontrolled point‐of‐care mobile photographs, along with variations in validation strategies (such as image‐level vs*.* our strict patient‐level stratified splitting), inherently shift the performance boundaries. Consequently, Table 9 serves as a descriptive benchmarking reference rather than an absolute indicator of architectural superiority.

An important consideration in interpreting the framework's diagnostic profiles is the inherent presence of label uncertainty. Within our multi‐source dataset, 55.3% (*n* = 390) of the images (primarily encompassing the medical atlas samples and confirmed institutional cases) possessed definitive histopathological confirmation, while the remaining 44.7% (*n* = 315) were diagnosed solely on clinical grounds via strict multi‐expert consensus. In deep learning, training on datasets with mixed diagnostic anchors can introduce minor label noise, which potentially bounds the model's ability to delineate exact micro‐architectural boundaries. This uncertainty most notably impacts visually confounding classes like Leukoplakia and Oral Lichen Planus (OLP), where hyperkeratotic overlaps can artificially cap single‐network precision. However, our reliance on a Multi‐Architecture Weighted Ensemble substantially mitigates this variance by aggregating complementary spatial and textural features, thereby stabilizing predictions even when confronted with borderline phenotypes induced by clinical label boundaries.

From a clinical perspective, the utility of a screening tool is governed not just by overall accuracy, but by the cost of errors. In oral cancer screening, a False Negative (missing a malignant lesion) is far more detrimental than a False Positive (biopsying a benign lesion). Our ensemble model achieved a Recall (Sensitivity) of 0.906 (95% CI: 88.4%–92.8%) for the overall test framework, which provides a critical safety margin. This stable sensitivity ensures that the system acts as a reliable screening adjunct, flagging potentially malignant cases for specialist review, while the controlled false‐positive rate ensures that the burden of unnecessary referrals remains manageable. However, the exceptional classification performance achieved for the Erythroplakia class (F1‐Score: 0.92) must be interpreted with caution due to the retrospective reliance on medical atlas sources for this rare malignancy. To mitigate the risk of the network adapting to image acquisition artifacts rather than true pathological features, strict ROI cropping was enforced, though validation on larger, multi‐institutional prospective cohorts remains necessary to confirm these stable performance profiles.

Beyond statistical metrics, the clinical adoption of AI systems demands transparency. To address the “black box” concern, we employed Grad‐CAM analysis. While the generated visual heatmaps qualitatively align with anatomical margins defined by clinicians, the current lack of localized quantitative metrics (e.g., pixel‐level IoU bounds) means these saliency maps should be interpreted strictly as an exploratory, qualitative clinical sanity check rather than a quantified proof of feature alignment. We explicitly acknowledge that without objective metrics, there is a substantial risk of overinterpreting these visualizations, as they do not definitively verify that the model's internal reasoning aligns with true clinical pathology. Furthermore, the Decision Curve Analysis (DCA) demonstrated the system's potential clinical value (Figure 6). Although the ensemble model yielded a higher simulated “Net Benefit” compared to standard “Treat‐All” and “Treat‐None” strategies across a wide range of threshold probabilities (0.1–0.9), this retrospective calculation must strictly not be interpreted as evidence of proven clinical utility.

To bridge the gap between retrospective net benefit calculations and proven clinical value, these threshold probabilities ($p_{t}$) can be directly mapped to distinct clinical triage pathways in primary dental care. Specifically, a low‐risk threshold ($p_{t}\;\leq$ 25%) corresponds to conservative observation and follow‐up. A moderate‐risk threshold (25%$\;<\;p_{t}\leq$ 60%) triggers a mandatory specialist consultation. Finally, a high‐risk threshold ($p_{t}\;>$ 60%) represents an urgent endpoint demanding an immediate referral for scalpel biopsy. By demonstrating a superior simulated Net Benefit across this spectrum, the DCA implies that utilizing the Weighted Ensemble could optimize the clinical triage process without artificially increasing unnecessary referrals, though prospective clinical trials are mandatory to establish true clinical utility.

Finally, the computational efficiency of the proposed ensemble supports its potential long‐term feasibility as a screening adjunct in resource‐constrained primary care settings. Despite aggregating three architectures, the system maintains an average inference time of 18 ms per image. This low latency implies that the model can function in near real‐time on standard medical workstations, facilitating immediate feedback during patient examinations without disrupting the clinical workflow. However, we emphasize that these computational and retrospective tracking advantages represent a preliminary technical feasibility. Actual deployment in primary dental care remains a future objective, pending rigorous external dataset validation, multi‐center prospective trials, and formal clinician‐in‐the‐loop interaction studies to ensure safety and usability.

## 5 Limitations

Despite the promising evaluation metrics achieved by the proposed framework, several major methodological limitations must be transparently disclosed to ensure a balanced interpretation of our results and potential clinical applicability.

First, the total dataset size (705 images) is relatively constrained compared to general computer vision benchmarks. While the cohort was rigorously curated and split using a strict stratified patient‐level protocol to prevent any internal data leakage, the independent test set remains small, containing only 63 images. Given the 5‐class nature of our classification task, performance estimates are based on a limited number of cases per category (e.g., 12 Normal, 10 Candidiasis, 13 Erythroplakia, 12 OLP, and 16 Leukoplakia). Consequently, small fluctuations in correctly or incorrectly classified samples could substantially alter the reported class‐wise metrics, warranting a cautious interpretation of statistical stability until evaluated on larger cohorts.

Second, there is an absence of definitive histopathological confirmation for 44.7% (*n* = 315) of the dataset, which was diagnosed solely on clinical grounds via expert consensus. Because our ground truth relied primarily on the multi‐expert clinical consensus of three independent board‐certified specialists, the training labels represent a “visual and clinical ground truth” rather than an absolute histological endpoint. Although strict inclusion criteria, requiring unanimous expert agreement and excluding ambiguous cases, were strictly enforced to minimize label noise, conditions like Leukoplakia and OLP can occasionally exhibit overlapping visual phenotypes that only biopsy can conclusively resolve. Consequently, it must be explicitly acknowledged that for these cases, the network is fundamentally learning to replicate expert clinical opinion rather than definitive true disease status. This inherent label uncertainty inevitably introduces a degree of label noise during model training, which may artificially bound the reported performance metrics and limit the ultimate precision of the network when evaluating these visually confounding classes. However, since the primary objective of this AI framework is to serve as a preliminary visual screening.

Third, the blend of clinical photographs with high‐quality medical atlas and textbook images introduces a potential domain shift. Textbook illustrations often showcase idealized, “textbook” lesion morphologies under optimal illumination and focal characteristics, which systematically differ from uncontrolled point‐of‐care photographs taken in routine dental practices. This limitation is particularly relevant to the Erythroplakia class, which achieved an exceptional F1‐score of 0.92 but relied heavily on retrospective atlas sources due to the very low clinical prevalence of this rare malignancy. While our preprocessing pipeline (Z‐score contrast normalization) aimed to homogenize the input space, and post‐hoc Grad‐CAM visualizations qualitatively verified that the network's attention was directed at the actual erythematous mucosal alterations rather than image source artifacts, the current lack of a source‐stratified performance analysis remains a constraint. To specifically address this, our newly incorporated source‐stratified evaluation confirmed that while a slight performance advantage exists for non‐clinical images (F1‐score: 93.4%) due to optimal resolution, the model still yields a robust F1‐score (89.1%) on unconstrained clinical photographs. Nevertheless, the reliance on reference sources for rare conditions like Erythroplakia remains a limitation, underscoring the absolute necessity for future training on large‐scale, multi‐institutional clinical cohorts.

Fourth, a significant pre‐analytical bias is introduced by the reliance on manual Region‐of‐Interest (ROI) cropping performed by experts prior to model training. Providing the multi‐architecture network with pre‐localized, tightly bounded lesions artificially simplifies the classification task compared to real‐world clinical workflows, where abnormalities are not pre‐annotated. Consequently, our reported metrics evaluate the model's capability to differentiate pre‐identified lesions rather than its capacity to actively detect them within uncropped, complex clinical oral cavity photographs. Future iterations must integrate an automated localization or object detection module (e.g., utilizing bounding‐box networks like YOLO) prior to the classification ensemble to enable a fully end‐to‐end automated screening pipeline.

Fifth, the explainability analysis utilizing Grad‐CAM is entirely qualitative and subjective. Although the generated visual heatmaps appear visually plausible and align with the pathognomonic margins defined by clinicians, no localized quantitative metrics were performed. Therefore, these visual saliency maps should be interpreted strictly as an exploratory qualitative clinical sanity check rather than quantified proof of consistent architectural feature alignment. We must critically emphasize that these heatmaps do not objectively verify that the model is learning clinically meaningful features, and extreme caution must be exercised to avoid overinterpreting these subjective explainability results.

Sixth, the present evaluation pipeline lacks a direct empirical comparison between the proposed Weighted Ensemble's predictions and the diagnostic performance of frontline clinical practitioners, such as general dentists, oral medicine specialists, or oral pathologists. Without this essential human benchmark, it remains difficult to determine the true practical significance and real‐world applicability of the reported performance metrics. While the model's metrics benchmark favorably against existing automated state‐of‐the‐art frameworks, validating its comparative accuracy and triage efficiency against human practitioners in blind reader studies is an unfulfilled step. Conducting such human‐vs‐AI benchmarking is vital to precisely quantify the framework's added diagnostic value as an interactive Clinical Decision Support System before field implementation.

Finally, a fundamental limitation of the present work is the complete absence of an independent, external validation dataset. Even though our strict patient‐level splitting rigorously prevented internal data leakage, evaluating the model solely on a unified curated dataset cannot guarantee generalizability to independent external institutions, highly diverse populations, or variations in point‐of‐care camera hardware and dynamic clinical workflows. Consequently, it must be explicitly acknowledged that it remains entirely unknown whether the framework would maintain its current diagnostic performance when deployed outside our specific institutional acquisition settings. To overcome the retrospective nature of this data collection, large‐scale, multi‐center prospective validation trials utilizing independent external cohorts are mandatory to definitively establish the true clinical robustness, generalizability, and real‐world impact of the proposed system on patient triage outcomes.

## 6 Conclusion

This study successfully developed and validated a Multi‐Architecture Weighted Ensemble Framework for the automated classification of pre‐localized high‐risk oral red‐white lesions from clinically confounding inflammatory conditions. By integrating the complementary strengths of ResNet‐50, Xception, and EfficientNet‐B0, the proposed system achieved a testing accuracy of 91.2%, outperforming individual baseline architectures and effectively addressing the persistent diagnostic challenge of distinguishing Oral Lichen Planus from premalignant Leukoplakia. Beyond raw classification performance, the qualitative integration of Grad‐CAM heatmaps and simulated Decision Curve Analysis (DCA) provided critical transparency regarding the model's exploratory clinical potential. However, while our low inference latency of 18 ms per image highlights strong technical efficiency, these retrospective results must be interpreted strictly as a preliminary proof‐of‐concept investigation rather than a clinically deployable system. Implementing this framework as a real‐time clinical decision support system in primary dental care cannot yet be recommended until it undergoes extensive external validation on independent institutional cohorts, rigorous prospective evaluation in true clinical screening environments, formal clinician‐in‐the‐loop interaction studies, and training on multi‐center datasets with definitive histopathological confirmation for all cases.

## Author Contributions

Mahsa Koochaki, Amirreza Hendi, and Hossein Sadr conceived of the presented idea. Amirreza Mousavie, Mojdeh Nazari, and Maryam Basirat collected the dataset, developed the theory, and performed the computations. Hossein Sadr and Mahsa Koochaki conceived the study and were in charge of overall direction and planning. Amirreza Mousavie and Maryam Basirat verified the analytical methods and obtained results. All authors discussed the results and contributed to the final manuscript.

## Funding

The authors have nothing to report.

## Disclosure

During the preparation of this work, the authors used the Gemini chatbot to improve the readability and language of the study. After using this tool, the authors reviewed and edited the content as needed and take full responsibility for the content of the published article.

## Ethics Statement

The project was found to be under the ethical principles and the national norms and standards for conducting Medical Research in Iran (Approval ID: IR.GUMS.REC.1403.176).

## Conflicts of Interest

The authors declare no conflicts of interest.

## Data Availability

The data that support the findings of this study are available on request from the first author. The data are not publicly available due to privacy or ethical restrictions. (Mahsa Koochaki, mahsakoochaki18@gmail.com).
